# Health Care Utilization and Costs for Older Adults Aging Into Medicare After the Affordable Care Act

**DOI:** 10.1001/jamahealthforum.2024.5025

**Published:** 2025-01-17

**Authors:** Renuka Tipirneni, Eric T. Roberts, Helen G. Levy, Andrei R. Stefanescu, Kenneth M. Langa, Kara Zivin, Donovan T. Maust, John Z. Ayanian

**Affiliations:** 1 Department of Internal Medicine, University of Michigan, Ann Arbor; 2 Institute for Healthcare Policy and Innovation, University of Michigan, Ann Arbor; 3 Department of Health Management and Policy, School of Public Health, University of Michigan, Ann Arbor; 4 University of Pennsylvania Perelman School of Medicine, Philadelphia; 5 Institute for Social Research, University of Michigan, Ann Arbor; 6 Gerald R. Ford School of Public Policy, University of Michigan, Ann Arbor; 7 Veterans Affairs Center for Clinical Management Research, Ann Arbor, Michigan; 8 Department of Psychiatry, University of Michigan, Ann Arbor; 9 Editor, *JAMA Health Forum*

## Abstract

**Importance:**

The Affordable Care Act (ACA) expanded Medicaid and Marketplace insurance to nonelderly adults in 2014, but whether these policies improved outcomes later in life is unknown.

**Objective:**

To examine whether exposure to ACA expansions during middle age (50-64 years) was associated with changes in health, utilization, and spending after these adults entered Medicare at 65 years of age.

**Design, Setting, and Participants:**

This serial analysis of the Health and Retirement Study cohort linked to Medicare enrollment and claims data from January 1, 2010, to December 31, 2018. Adults aged 65 to 68 years entering Medicare after the ACA (exposed to ACA expansions during middle age) were compared with adults entering Medicare before the ACA (4452 person-years). Interrupted time series analyses were used to assess overall changes associated with exposure to ACA expansions and difference-in-differences analyses to isolate changes associated with Medicaid expansion among low-income adults (incomes ≤400% of the federal poverty level for any ACA coverage and ≤138% for Medicaid expansion coverage). Data were analyzed from March 1, 2023, to May 1, 2024.

**Exposures:**

ACA coverage expansion overall in 2014 and Medicaid expansion as of 2018.

**Main Outcomes and Measures:**

Health (self-reported overall, activities of daily living [ADL], instrumental ADL, and depressive symptoms), utilization (outpatient visits, emergency department visits, and hospital admission), and costs (self-reported out-of-pocket and Medicare costs).

**Results:**

Among the analytic sample of 2782 participants (mean age, 66.4 [95% CI, 66.3-66.5] years), a weighted 59.1% (95% CI, 55.3%-62.7%) were female. In interrupted time series analyses, reductions across cohorts were found in use of chronic disease medications (−5.0 [95% CI, −9.8 to −0.3] percentage points), hospitalizations per year (−0.2 [95% CI, −0.4 to −0.03]), and out-of-pocket costs (−$417 [95% CI, −$694 to −$139]) but no significant changes across cohorts in health status, outpatient or emergency visits, or Medicare costs. In difference-in-differences analyses relative to nonexpansion states, greater reductions were found in the number of ADL limitations (−0.4 [95% CI, −0.8 to −0.02]) and lesser reductions in out-of-pocket costs ($900 [95% CI, $275-$1526]) in Medicaid expansion states but otherwise similar changes in other outcomes.

**Conclusions and Relevance:**

This study found modest evidence of reductions in out-of-pocket costs and improvements in health among adults entering Medicare after the ACA. Insurance coverage and financial assistance should be preserved and enhanced to improve health and health care access among vulnerable older adults.

## Key Points

**Question:**

Is exposure to Affordable Care Act (ACA) expansions during middle age associated with improvements in health, utilization, and spending after these adults enter Medicare at 65 years of age?

**Findings:**

This study analyzed the Health and Retirement Study and Medicare claims data of 2782 participants and found reductions in medication use for chronic disease, hospitalizations, and out-of-pocket costs among cohorts entering Medicare after vs before the ACA. In Medicaid expansion vs nonexpansion states, greater reductions in activities of daily living limitations and lesser reductions in out-of-pocket costs occurred.

**Meaning:**

These findings suggest that insurance coverage and financial assistance should be preserved and enhanced to improve health and health care access among vulnerable older adults.

## Teaser

This cohort study assesses changes in overall health, health care utilization, and costs for patients gaining Medicare coverage before and after Medicaid expansion and availability of Marketplace insurance plans through the Affordable Care Act.

## Introduction

US middle-aged adults have experienced worsening morbidity and mortality over the past decade.^1,2^ Case and Deaton^3,4,5^ examined drivers of this phenomenon and have described increased distress, morbidity, and premature “deaths of despair” due to drug and alcohol use, suicide, and cardiometabolic diseases among middle-aged adults with low socioeconomic status. Mirroring data on premature mortality,^6,7^ US middle-aged adults also experienced declines in daily health-related functioning,^8^ concentrated among individuals with limited educational attainment and low incomes.^2^ This worsening of health outcomes was exacerbated by the COVID-19 pandemic.^9^

Health insurance has been associated with improved health outcomes in the general population and among older adults gaining Medicare coverage at 65 years of age prior to the Affordable Care Act (ACA).^10,11,12,13^ Following the ACA’s large growth in health insurance coverage in 2014 via Medicaid expansion (for individuals with incomes ≤138% of the federal poverty level [FPL] in states that expanded) and private Marketplace plans (with financial assistance through tax credits and cost-sharing subsidies for individuals with incomes 100%-400% of the FPL in all states), numerous studies have found improvements in access to care and health among nonelderly adults.^8,14,15^

However, few studies have examined the impact of the ACA on health and financial outcomes among middle-aged (50-64 years) or older (≥65 years) adults,^16,17,18,19^ despite their greater burdens of chronic health conditions and financial strain from medical costs. Earlier studies found reduced mortality among middle-aged adults both related to Medicaid expansion and to tax credits for private Marketplace plans under the ACA.^19,20^ As these mortality reductions occurred predominantly among middle-aged adults, it is important to understand the potential mechanisms through which ACA coverage expansions affect health for this critical age group, such as earlier detection and management of chronic conditions. In addition, many prior studies of older adults were single cross-sectional analyses and limited in assessing the association of exposures in one period (ACA coverage eligibility in midlife) with outcomes in a later period (after Medicare eligibility at 65 years of age).

One prior longitudinal analysis of middle-aged adults with low socioeconomic status conducted in 2010 to 2016^17^ found that ACA Medicaid expansion led to greater insurance coverage and also increased hospitalizations after 2 years. This increase in hospitalizations was possibly related to pent-up demand for care and newly diagnosed health conditions. Previous studies have shown evidence of pent-up demand for care on reaching Medicare eligibility at 65 years of age.^21^ However, whether the ACA, by expanding coverage and improving access to care prior to 65 years of age, subsequently improved health and reduced pent-up demand for care among new Medicare beneficiaries remains unknown.

For adults in their 50s and early 60s on the cusp of Medicare eligibility who had limited coverage options before the ACA, improved access to care in midlife might allow for earlier detection and management of health risks, improvements in health, and reductions in potentially preventable hospitalizations after entry to Medicare at 65 years of age. The potential for such longer-term benefits of health insurance are supported by prior studies that linked childhood Medicaid coverage to long-term improved health in adulthood.^22,23,24^ Furthermore, changes in need for and use of care might affect older adults’ health care spending and associated out-of-pocket costs at Medicare enrollment. Therefore, we examined whether exposure to the ACA’s coverage expansions among middle-aged adults led to changes in health, use of health care, and out-of-pocket costs after these adults enrolled in Medicare at 65 years of age.

## Methods

### Data Sources

We conducted serial cross-sectional analyses of the Health and Retirement Study (HRS) cohort linked to Medicare enrollment and claims data from January 1, 2010, to December 31, 2018. The HRS is a longitudinal, nationally representative survey of older US residents, conducted biennially in person and by telephone among people 51 years and older with response rates between 70% and 90%.^25^ As the HRS assesses self-reported income and health measures in every survey wave, income data are available for participants both prior to and after 65 years of age, allowing for identification of individuals who were eligible for ACA coverage in midlife and assessment of health outcomes after Medicare eligibility. Additionally, as 94% of respondents currently consent to the linkage of HRS survey data to Medicare enrollment data for all participants and claims data for fee-for-service beneficiaries, it is possible to assess key utilization outcomes after Medicare entry.^26^ The study was approved by the University of Michigan institutional review board without the need for informed consent due to use of deidentified data and followed the Strengthening the Reporting of Observational Studies in Epidemiology (STROBE) guidelines for cross-sectional and cohort studies.

### Study Population

We included cohorts of adults newly enrolling in Medicare (aged 65-68 years at the time of HRS interview) before (2010-2012) and after (2016-2018) the 2014 ACA coverage expansions. Cohorts entering Medicare from 2016 to 2018 had exposure to ACA expansions prior to becoming eligible for Medicare at 65 years of age (see study sample selection in the eMethods in Supplement 1). We focused on people with low incomes eligible for ACA coverage prior to Medicare entry (≤400% of the FPL for any ACA coverage and ≤138% of the FPL for Medicaid expansion coverage at 60-64 years of age). We excluded people with Medicare enrollment prior to 65 years of age. We also excluded residents of Wisconsin, which increased Medicaid eligibility for adults with incomes of 100% or less of the FPL but did not implement a full Medicaid expansion under the ACA.

### Measures

We examined 2 primary exposures. First, we assessed exposure to ACA coverage expansions overall (Marketplace and Medicaid) implemented in 2014. Second, we assessed exposure to ACA Medicaid expansion by comparing residents of states that did and did not expand Medicaid as of January 1, 2018 (eMethods in Supplement 1). Study participants who were younger than 65 years any time after 2014 were considered exposed to the expansions. Six states that expanded Medicaid early (prior to 2014) under the ACA still experienced further enrollment increases after 2014 and were thus classified as expansion states.

Our primary outcomes focused on health, health care utilization, and costs measured between 65 and 68 years of age. We used the following self-reported measures of health from the HRS survey: overall health status (dichotomized as excellent or very good vs good, fair, or poor as in prior literature)^27^; functional limitations, including activities of daily living (ADLs; count of difficulties with walking, dressing, bathing, eating, transferring, or toileting) and instrumental ADLs (IADLs; count of difficulties with cooking, grocery shopping, making phone calls, taking medications, or managing finances); and presence of depressive symptoms (dichotomized as ≥4 of 8 items on the Center for Epidemiological Studies Depression scale, as in other HRS studies).^28,29^ We also assessed self-reported use of prescription medications, which were dichotomized as any vs none, as the survey items assessed use of medications for a focused set of chronic conditions and could not provide a comprehensive count of medications.

We assessed health care utilization measures as counts per year in Medicare claims data. These measures included outpatient visits, emergency department (ED) visits, and inpatient hospital admission (eMethods in Supplement 1).

For out-of-pocket costs, we used self-reported measures assessing costs over the 2 years prior to the HRS interview, which we inflated to 2018 dollars using the Consumer Price Index. This self-reported HRS measure sums all major out-of-pocket medical costs, including costs related to hospital care, nursing home care, outpatient surgery, physician visits, dental care, prescription drugs, in-home health care, and other health services such as adult day care, social work, outpatient rehabilitation, physical therapy, and health-related transportation services over the preceding 2 years.

We determined Medicare costs from claims files with 2 separate measures: payments by the Medicare program, and total costs including contributions from Medicare and the beneficiary. In contrast to the HRS self-reported measure, this latter claims-based measure only accounts for the beneficiary’s contribution to deductibles and coinsurance per year (rather than over 2 years) and does not include copayments or other out-of-pocket expenses.

Covariates included self-reported sex, race and ethnicity, educational level, marital status, assets (inflated to 2018 US dollars using the Consumer Price Index), and year fixed effects. We included race and ethnicity to account for known differences in enrollment, due to structural racism and discrimination and to outreach and enrollment efforts that focused on underserved racial and ethnic groups. Categories of race and ethnicity were Hispanic, non-Hispanic Black, non-Hispanic White, and other (including non-Hispanic American Indian or Alaskan Native, non-Hispanic Asian, and non-Hispanic Native Hawaiian or Other Pacific Islander). We did not include age, given the narrow age band of our study cohorts and collinearity with study year. We also did not include state fixed effects, due to convergence issues in analysis.

### Statistical Analysis

Data were analyzed from March 1, 2023, to May 1, 2024. To assess overall changes following the ACA’s coverage expansions, we conducted interrupted time-series (ITS) analyses comparing outcomes between older adult cohorts in the years before (comparison cohort of adults aged 65-68 years whose entry into Medicare preceded the ACA) and after (treatment cohort of adults aged 65-68 years in 2016-2018 and thus exposed to ACA coverage expansions prior to turning 65 years of age) implementation in 2014. The main explanatory variables included indicators for the 2016 and 2018 periods, which characterized the mean change in an outcome from baseline through each post-ACA period. Most state Medicaid expansions under the ACA started in 2014, and the federal Marketplace became fully operational by early 2014 (eMethods in Supplement 1). We expected at least a 1-year lag in the effects of the ACA on health and utilization outcomes; thus, we considered 2018 the primary postexposure year.

To isolate effects associated with Medicaid expansion specifically, we conducted difference-in-differences (DID) analyses comparing changes between exposed (post-ACA) and unexposed (pre-ACA) cohorts in states that expanded Medicaid by January 2018 with states that did not. The main explanatory variables were interactions between residence in a Medicaid expansion state and indicators for 2016 and 2018 postexpansion years. As most states in the study period expanded in 2014 to 2015, we considered the 2014 HRS wave the change year for all DID analyses. To account for lagged effects, we focused on DID estimates characterizing changes between the pre-ACA and the 2018 post-ACA cohort between expansion vs nonexpansion states.

We assessed for parallel trends between expansion and nonexpansion states in the pre-expansion period and found this assumption satisfied for all outcomes except outpatient visits, Medicare payments, and total Medicare costs (eMethods in Supplement 1). We used logistic regression models for binary variables (self-reported health, presence of depressive symptoms, and prescription medication use), negative binomial regression for count variables (ADLs, IADLs, and outpatient visits), zero-inflated negative binomial regression for rare outcomes (ED visits and hospitalizations), and linear regression for log-transformed cost variables (out-of-pocket costs, Medicare costs, and total costs).

All analyses used survey weights and adjusted for the covariates noted above. We estimated marginal probabilities to report point estimates and adjusted percentage point changes with 95% CIs in all ITS and DID models. This approach to marginal estimation accounts for the fact that estimates in nonlinear models depend on the values of other covariates in the model.^30^

We conducted sensitivity analyses excluding (1) the 6 states that expanded Medicaid early (before 2014) under the ACA and (2) the 2 states that expanded Medicaid later in 2016. To interpret the magnitude of health and utilization changes after Medicare entry in the context of coverage changes for middle-aged adults, we conducted a separate analysis that demonstrated large coverage gains for individuals aged 50 to 64 years (eMethods in Supplement 1). We used Stata, version 17.0 (StataCorp LLC), for all analyses. The threshold for statistical significance was set at 2-tailed *P* < .05.

## Results

### Characteristics of HRS Participants

Our final HRS analytic sample included 2782 low-income older adults (4452 person-years) who newly entered Medicare after 65 years of age (2358 person-years in Medicaid expansion states and 2418 person-years in nonexpansion states). For the linked Medicare claims analyses, our cohort included 938 older adults or 1214 person-years (590 person-years in expansion states and 624 person-years in nonexpansion states; mean age, 66.4 [95% CI, 66.3-66.5] years). At baseline, a weighted 59.1% (95% CI, 55.3%-62.7%) of older adults identified as female and 40.9% (95% CI, 37.3%-44.7%) as male. In terms of race and ethnicity, 10.8% (95% CI, 6.7%-16.8%) identified as Hispanic; 10.8% (95% CI, 8.7%-13.3%), non-Hispanic Black; 75.7% (95% CI, 70.6%-80.1%), non-Hispanic White; and 2.8% (95% CI, 1.7%-4.8%), other. For educational attainment. 57.5% (95% CI, 52.0%-62.9%) reported having a high school education or below, 24.0% (95% CI, 20.0%-28.9%) reported some college, and 18.5% (95% CI, 15.0%-22.5%) reported having a college degree or above (Table 1).

**Table 1.** Baseline Characteristics of Health and Retirement Study Participants Aged 65 to 68 Years With Income up to 400% of the FPL, 2012^a^

Characteristic	Survey-weighted % (95% CI)
Age, mean (95% CI), y	66.4 (66.3-66.5)
Sex	
Female	59.1 (55.3-62.7)
Male	41.9 (37.3-44.7)
Race and ethnicity	
Hispanic	10.8 (6.7-16.8)
Non-Hispanic Black	10.8 (8.7-13.3)
Non-Hispanic White	75.7 (70.6-80.1)
Other^b^	2.8 (1.7-4.8)
Educational level	
High school or below	57.5 (52.0-62.9)
Some college	24.0 (20.0-28.9)
College and above	18.5 (15.0-22.5)
Income, FPL group	
≤138%	31.6 (26.9-36.7)
139%%-250%	30.4 (26.3-34.8)
251%-400%	38.1 (33.8-42.5)
Total assets, median (95% CI), 2018 US $	358 682 (286 945-430 419)
Married	58.8 (54.7-62.7)

Abbreviation: FPL, federal poverty level.

^a^ All estimates incorporate Health and Retirement Study complex survey weights.

^b^ Includes non-Hispanic American Indian or Alaska Native, non-Hispanic Asian, and non-Hispanic Native Hawaiian or Other Pacific Islander.

### Overall Changes in Health, Utilization, and Costs Before and After the ACA

Relative to adults aged 65 to 68 years who entered Medicare before the ACA (comparison cohort), those entering Medicare after the ACA (ie, treatment cohort exposed to ACA coverage in midlife) were less likely to report using medications for chronic disease (−5.0 [95% CI, −9.8 to −0.3] percentage points), had fewer hospitalizations per year (mean, −0.2 [95% CI, −0.4 to −0.03]), and had reduced self-reported out-of-pocket costs over 2 years (mean, −$417 [95% CI, −$694 to −$139]) (Table 2). However, no significant differences were noted in health status, outpatient or ED visits, or Medicare costs between treatment and comparison cohorts (Table 2). Outcome trends over the study period are depicted in the Figure.

**Table 2.** Interrupted Time Series Analysis of Overall Changes in Health, Utilization, and Costs for Adults Aged 65 to 68 Years With Income up to 400% of the Federal Poverty Level^a^

Outcome	Pre ACA (95% CI)	Post ACA (95% CI)	Change (95% CI)	*P* value
Health status^b^				
Excellent or very good health, %	41.6 (37.8 to 45.4)	42.0 (38.2 to 45.8)	0.4 (−4.8 to 5.5)	.88
Depressive symptoms, %	12.7 (9.5 to 15.9)	10.2 (7.2 to 13.2)	−2.5 (−6.7 to 1.8)	.25
No. of ADL limitations	0.3 (0.2 to 0.4)	0.3 (0.2 to 0.4)	0.0 (−0.1 to 0.2)	.71
No. of IADL limitations	0.2 (0.1 to 0.3)	0.2 (0.1 to 0.2)	0.0 (−0.1 to 0.0)	.28
Utilization^c^				
Using medication for chronic conditions, %	84.4 (81.1 to 87.7)	79.4 (75.9 to 82.9)	−5.0 (−9.8 to −0.3)	.04
Total No. of outpatient visits per year	1.2 (0.8 to 1.6)	0.8 (0.5 to 1.2)	−0.4 (−0.9 to 0.2)	.18
Total No. of ED visits per year	0.5 (0.2 to 0.7)	0.4 (0.2 to 0.6)	−0.1 (−0.3 to 0.2)	.59
Total No. of hospital admissions per year	0.3 (0.2 to 0.5)	0.1 (0.1 to 0.2)	−0.2 (−0.4 to 0.0)	.03
Out-of-pocket costs over 2 y, US $^b^	1646 (1431 to 1861)	1230 (1064 to 1395)	−417 (−694 to −139)	.004
Medicare costs, mean, US $^c^				
Medicare payment per year	2476 (1709 to 3243)	1742 (1130 to 2354)	−734 (−1774 to 305)	.16
Total cost including beneficiary and Medicare payments per year	2791 (1952 to 3629)	2075 (1425 to 2725)	−716 (−1794 to 363)	.19

Abbreviations: ACA, Affordable Care Act; ADL, activities of daily living; ED, emergency department; IADL, instrumental ADL.

^a^ Changes represent the difference between the baseline year (2012) and the final year available for analysis (2018) based on ACA’s insurance coverage expansions in 2014. All analyses were survey weighted and adjusted for sex, race and ethnicity, educational level, marital status, and assets, as well as year fixed effects.

^b^ From Health and Retirement Study core survey data.

^c^ From linked fee-for-service Medicare claims data.

**Figure.**
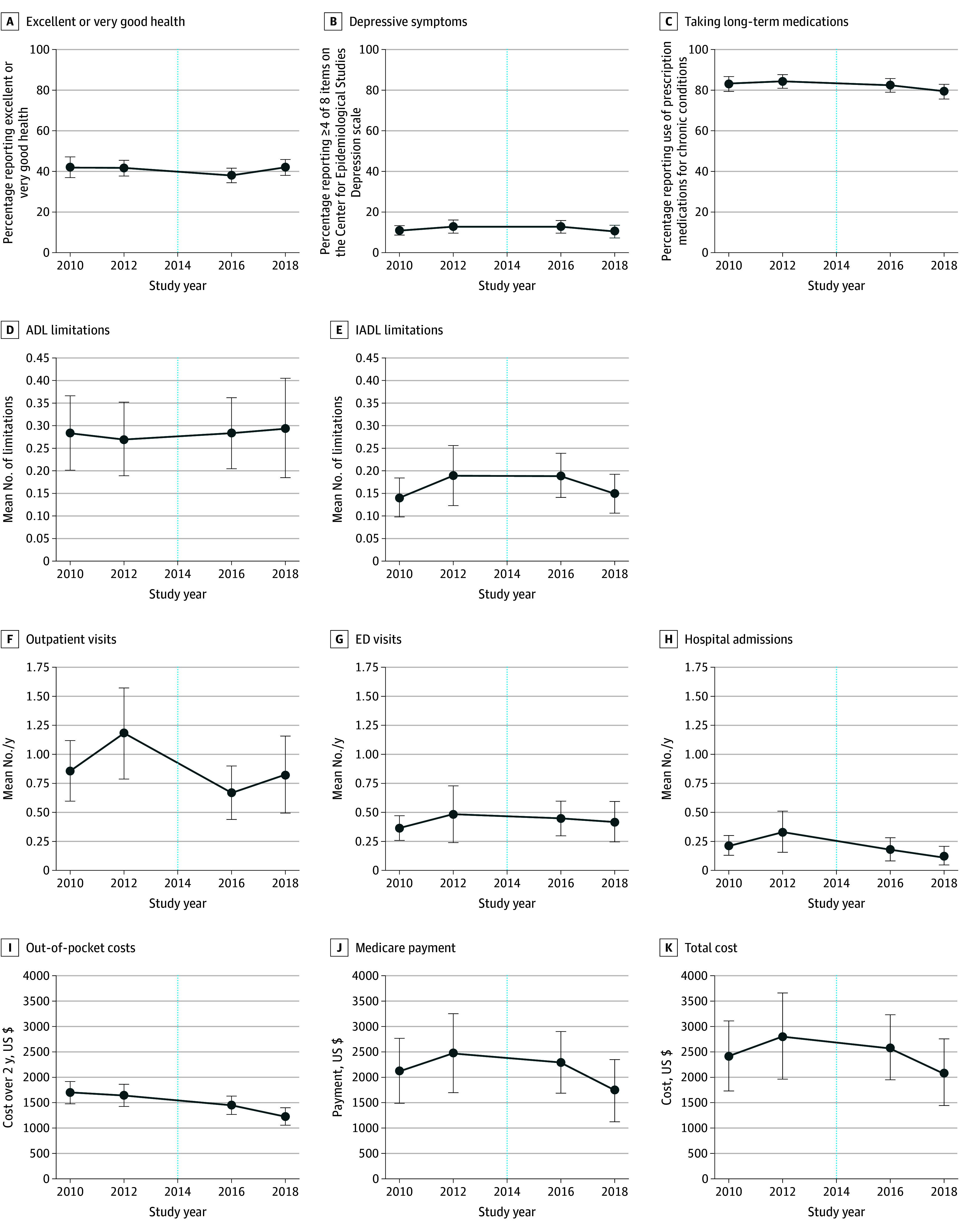
Trends in Health, Utilization, and Costs for Adults Aged 65 to 68 Years With Income up to 400% of the Federal Poverty Level Before and After the Affordable Care Act Interrupted time series analysis was used to assess overall changes before compared with after the Affordable Care Act’s insurance coverage expansions in 2014, which is indicated in each graph by a dotted line. All analyses were survey weighted and adjusted for sex, race and ethnicity, educational level, marital status, and assets, as well as year fixed effects. Error bars represent 95% CIs. ADL indicates activities of daily living; ED, emergency department; and IADL, instrumental ADL.

### Differential Changes in Medicaid Expansion States

In DID analyses comparing cohorts of adults aged 65 to 68 years who were previously exposed vs unexposed to ACA coverage expansions, we found greater reductions in self-reported activities of daily living limitations (DID, −0.4 [95% CI, −0.8 to −0.02] limitations) and lesser reductions in self-reported out-of-pocket costs (DID, $900 [95% CI, $275-$1526]) in Medicaid expansion states relative to nonexpansion states (Table 3). Otherwise, we observed similar changes for older adults in Medicaid expansion compared with nonexpansion states in other measures of health status, self-reported prescription medication use, outpatient and ED visits, hospitalizations, and Medicare costs (Table 3).

**Table 3.** Difference-in-Difference Analyses Comparing Changes for Adults Aged 65 to 68 Years With Income up to 138% of the Federal Poverty Level in Medicaid Expansion and Nonexpansion States^a^

Outcome	Expansion states			Nonexpansion states			DID (95% CI)^b^	*P* value^b^
	Pre ACA	Post ACA	Change	Pre ACA	Post ACA	Change		
Health status^b^								
Excellent or very good health, %	31.5 (23.6 to 39.4)	39.1 (32.9 to 45.3)	7.6 (−1.3 to 16.5)	31.8 (22.5 to 41.1)	34.6 (27.3 to 41.8)	2.8 (−7.7 to 13.2)	4.8 (−7.9 to 17.6)	.45
Depressive symptoms, %	20.4 (11.1 to 29.8)	14.4 (8.2 to 20.6)	−6.0 (−17.6 to 5.6)	17.4 (8.8 to 26.1)	17.7 (7.9 to 27.5)	0.2 (−9.9 to 10.3)	−6.2 (−18.8 to 6.3)	.32
No. of ADL limitations	0.5 (0.2 to 0.9)	0.4 (0.2 to 0.6)	−0.2 (−0.5 to 0.2)	0.4 (0.2 to 0.6)	0.6 (0.3 to 0.9)	0.3 (−0.1 to 0.6)	−0.4 (−0.8 to 0.0)	.04
No. of IADL limitations	0.3 (0.1 to 0.5)	0.2 (0.1 to 0.3)	−0.1 (−0.2 to 0.1)	0.3 (0.1 to 0.5)	0.4 (0.3 to 0.6)	0.2 (−0.1 to 0.4)	−0.2 (−0.5 to 0.1)	.11
Utilization^b^								
Use of medication for chronic conditions, %	80.4 (72.3 to 88.5)	76.3 (69.6 to 83.0)	−4.1 (−13.9 to 5.7)	86.9 (80.7 to 93.1)	85.2 (79.2 to 91.3)	−1.7 (−10.7 to 7.4)	−2.4 (−12.6 to 7.8)	.64
Total No. of outpatient visits per year	1.2 (0.7 to 1.8)	1.4 (0.7 to 2.2)	0.2 (−0.8 to 1.2)	1.5 (0.4 to 2.5)	1.1 (0.6 to 1.6)	−0.4 (−1.3 to 0.6)	0.5 (−0.7 to 1.8)	.39
Total No. of ED visits per year	0.3 (0.1 to 0.6)	0.8 (0.4 to 1.1)	0.4 (0.0 to 0.8)	0.6 (0.0 1.1)	0.4 (0.1 to 0.6)	−0.2 (−0.8 to 0.4)	0.6 (0.0 to 1.3)	.06
Total No. of hospital admissions per year	0.2 (0.1 to 0.3)	0.2 (0.0 to 0.3)	0.0 (−0.2 to 0.2)	0.6 (−0.4 to 1.6)	0.2 (0.0 to 0.4)	−0.4 (−1.3 to 0.5)	0.4 to (−0.6 to 1.3)	.43
Out-of-pocket costs over 2 y, US $^b^	1440 (1013 to 1867)	1291 (986 to 1595)	−149 (−615 to 316)	2144 (1641 to 2647)	1094 (796 to 1392)	−1050 (−1680 to −420)	900 (275 to 1526)	.006
Medicare costs, US $^c^								
Medicare payment per year	2472 (1121 to 3823)	3102 (1528 to 4675)	630 (−1321 to 2581)	2822 (1154 to 4489)	3467 (1512 to 5421)	645 (−1870 to 3160)	−15 (−2929 to 2899)	.99
Total cost including beneficiary and Medicare payments per year	2274 (1242 to 4205)	3267 (1607 to 4926)	543 (−1528 to 2614)	3100 (1244 to 4956)	3524 (1499 to 5548)	424 (−2216 to 3064)	119 (−2941 to 3179)	.94

Abbreviations: ACA, Affordable Care Act; ADL, activities of daily living; DID, difference-in-difference; ED, emergency department; FPL, federal poverty level; IADL, instrumental ADL.

^a^ Difference-in-difference analyses were used to compare changes in states that expanded Medicaid under the Affordable Care Act from the baseline year (2012) and the final year available for analysis as of January 1, 2018, with states that did not expand Medicaid. All analyses were survey weighted and adjusted for sex, race and ethnicity, marital status, educational level, and assets, as well as year fixed effects.

^b^ From Health and Retirement Study core survey data.

^c^ From linked fee-for-service Medicare claims data.

### Sensitivity Analyses

To assess the robustness of our findings, we conducted sensitivity analyses excluding 6 states that expanded Medicaid early under the ACA from analyses (eTables 1 and 2 in Supplement 1) and 2 states that expanded Medicaid after 2014 to 2015 (eTables 3 and 4 in Supplement 1). In all analyses, results remained similar to those of the primary analyses.

## Discussion

In this study of older US adults, we found that those who turned 65 years of age and aged into Medicare after the 2014 ACA coverage expansions (ie, exposed to these expansions in midlife) compared with those who aged in before the ACA had significantly lower self-reported out-of-pocket costs, self-reported chronic disease medication use, and rates of hospitalization. The latter finding suggests that exposure to ACA coverage in midlife could have contributed to prevention or better management of chronic health conditions that drive health care use later in life. When comparing cohorts of low-income older adults exposed vs unexposed to ACA policies in midlife, those residing in Medicaid expansion states had greater reductions in activities of daily living limitations and lesser reductions in out-of-pocket costs after 65 years of age, but otherwise similar changes in outcomes compared with those in nonexpansion states. Overall, these findings demonstrate some reductions in out-of-pocket costs and hospitalizations among adults entering Medicare after the ACA, as well as modest improvements in functional status among people exposed to Medicaid expansion in midlife.

Our findings of long-term benefits of coverage expansion over the life course build on previous studies examining early-life exposure to changes in insurance eligibility.^22,23,24,31,32,33^ For example, prior studies^22,31^ linked prenatal or early childhood Medicaid eligibility to fewer hospitalizations in adulthood, including potentially preventable hospitalizations. Other studies^23,24,32,33^ found that early-life Medicaid exposure was associated with long-term improvements in oral health, disability, and lower mortality, particularly for Black populations. While early childhood has long been considered a critical period that influences health, our findings suggest that midlife is another key period when insurance-related policy interventions may improve long-term health outcomes.

Our study of adults exposed to the ACA in midlife found similar health and financial benefits to prior studies of all nonelderly adults gaining coverage under the ACA.^8,14,15,19,34,35,36,37,38,39,40,41,42^ However, while we observed a decline in out-of-pocket spending among adults aging into Medicare after 2014, the reduction was less pronounced in Medicaid expansion states compared with nonexpansion states, in contrast to prior research linking Medicaid expansion with lower out-of-pocket spending among nonelderly adults.^14^ Several factors might account for these contrasting findings. As individuals age into Medicare, out-of-pocket costs are driven by changes in cost-sharing and utilization patterns on Medicare entry. Individuals transitioning from Medicaid (which has minimal cost sharing) may face significant changes in out-of-pocket expenses, depending on whether they continue to qualify for Medicaid or enroll in a Medicare Savings Program, each of which covers different Medicare costs. Further research is needed to understand mechanisms underlying these changes in out-of-pocket costs.

In addition, middle-aged adults may have benefitted similarly from Medicaid and nongroup private coverage obtained through ACA Marketplaces. In a prior study of middle-aged adults gaining coverage under the ACA,^17^ our group found that overall coverage rates increased similarly in Medicaid expansion and nonexpansion states, with larger gains in Medicaid coverage in expansion states and larger gains in private coverage in nonexpansion states. It is also possible that other features of the ACA, including nationwide cost-sharing reductions for certain preventive services, were particularly impactful for health and health care use among middle-aged adults, driving observed improvements after 2014, while the type of coverage may have been less influential on these outcomes in older adult populations. In addition, as out-of-pocket costs already appeared to be declining for older adults prior to 2014, it is possible that some reductions in out-of-pocket costs were associated with other factors such as the closing of the Medicare Part D “donut hole” under the ACA.

Our observation of overall reductions in self-reported out-of-pocket medical costs among older adults aging into Medicare after the ACA echo findings from the Oregon Health Insurance Experiment, which found near elimination of catastrophic out-of-pocket costs among nonelderly adults who were randomized to receive Medicaid coverage.^43^ While out-of-pocket costs declined for individuals living in both expansion and nonexpansion states in our study, we observed greater reductions in out-of-pocket costs in nonexpansion states, which may relate to differences in cost sharing on Medicare entry, as noted above. In addition, our finding of no change in Medicare costs among older adults entering Medicare eligibility differs from a prior study examining spillover effects of pre-ACA Medicaid expansions for working-age adults on Medicare beneficiaries.^44^ In that study, McInerney and colleagues^44^ found that pre-ACA Medicaid expansions to adults younger than 65 years were associated with small, contemporaneous reductions in Medicare spending among older adults with dual eligibility for Medicare and Medicaid.

### Policy Implications

This study suggests some key policy implications. First, the findings demonstrate significant associations of eligibility for Marketplace financial assistance with reductions in out-of-pocket costs and hospitalizations as adults age into older adulthood. Currently, enhanced Marketplace subsidies implemented under the Inflation Reduction Act of 2022 are set to expire at the end of 2025 and require Congressional action to sustain. Second, previous proposals have sought to lower the age of Medicare eligibility below 65 years, to allow middle-aged adults to maintain health insurance even when leaving the labor force. The findings suggest that the ACA expansions have provided a complementary set of coverage options for middle-aged adults in the years before Medicare eligibility.

### Strengths and Limitations

The HRS has several strengths, including use of a nationally representative sample with identification of income both before and after 65 years of age, which allowed us to identify people exposed to ACA coverage eligibility in midlife prior to aging into Medicare. However, smaller sample sizes in the HRS limit the statistical power of the study. With larger sample sizes, we may have been able to detect small but meaningful changes in health measures, which trended toward improvement but were not statistically significant in our analyses. Other study limitations include a focus on fee-for-service claims, which were the only Medicare claims available for assessment of utilization measures. Medicare Advantage enrollees represent a large and growing share of Medicare beneficiaries yet represented a minority (25%-37%) of enrollment in the study period.^45^ If individuals with lower incomes differentially selected Medicare Advantage over traditional fee-for-service Medicare, then that could have reduced our sample sizes for the claims analyses and biased our findings to the null. If ACA coverage expansions improved health and this resulted in more new Medicare enrollees choosing Medicare Advantage plans (reflecting positive selection into Medicare Advantage based on health),^46^ then that could also bias the claims analyses against finding true effects; however, the survey data would not have that potential bias. As prior studies have found reduced mortality after ACA expansions,^19,20^ if deaths became postponed among sicker people, this could attenuate observed effects on health and bias our health findings to the null.

For our ITS analyses, our analytic approach focused on comparing outcomes between different age cohorts with vs without exposure to ACA expansions (ie, turning 65 years of age after vs before 2014) and may be confounded by other time-varying factors that coincide with the ACA. The ACA also introduced several other coverage and cost-sharing reforms, such as a requirement for all insurers to cover preventive services with no cost sharing, though this provision should not have differentially impacted cohorts entering Medicare before vs after 2014. For our DID analyses, we did not account for staggered treatment timing, as in the study period most states expanded Medicaid in 2014 to 2015. We also conducted a sensitivity analysis excluding later-expanding states and found similar results.

## Conclusions

This study found evidence of some reductions in out-of-pocket costs and modest improvements in health among adults entering Medicare after the ACA. Insurance coverage and financial assistance should be preserved and enhanced to improve health and health care access among vulnerable middle-aged and older adults.

## Notes

Supplement 1. **eMethods.** Methodologic Appendix **eTable 1.** Interrupted Time Series: Overall Changes in Health, Utilization, and Costs for Adults Aged 65 to 68 Years With Income Up to 400% FPL Before and After the Affordable Care Act, Excluding Early Expansion States (n = 3745 Person-Years) **eTable 2.** Difference-in-Difference Analyses Comparing Changes for Adults Aged 65 to 68 Years With Income Up to 138% FPL in Medicaid Expansion and Nonexpansion States, Excluding Early Expansion States (n = 1074 Person-Years) **eTable 3.** Interrupted Time Series: Overall Changes in Health, Utilization, and Costs for Adults Aged 65 to 68 Years With Income Up to 400% FPL Before and After the Affordable Care Act, Excluding Montana and Louisiana (n = 4359 Person-Years) **eTable 4.** Difference-in-Difference Analyses Comparing Changes for Adults Aged 65 to 68 Years With Income Up to 138% FPL in Medicaid Expansion and Nonexpansion States, Excluding Montana and Louisiana (n = 1184 Person-Years)
eReference.

Supplement 2.
Data Sharing Statement
