# Supplementary material for: Health Care Utilization and Costs for Older Adults Aging Into Medicare After the Affordable Care Act
Source: JAMA Health Forum. 2025 Jan 17;6(1):e245025. doi: 10.1001/jamahealthforum.2024.5025 (PMC11742520; doi:10.1001/jamahealthforum.2024.5025)
Supplement: Supplement 1. — eMethods. Methodologic Appendix eTable 1. Interrupted Time Series: Overall Changes in Health, Utilization, and Costs for Adults Aged 65 to 68 Years With Income Up to 400% FPL Before and After the Affordable Care Act, Excluding Early Expansion States (n = 3745 Person-Years) eTable 2. Difference-in-Difference Analyses Comparing Changes for Adults Aged 65 to 68 Years With Income Up to 138% FPL in Medicaid Expansion and Nonexpansion States, Excluding Early Expansion States (n = 1074 Person-Years) eTable 3. Interrupted Time Series: Overall Changes in Health, Utilization, and Costs for Adults Aged 65 to 68 Years With Income Up to 400% FPL Before and After the Affordable Care Act, Excluding Montana and Louisiana (n = 4359 Person-Years) eTable 4. Difference-in-Difference Analyses Comparing Changes for Adults Aged 65 to 68 Years With Income Up to 138% FPL in Medicaid Expansion and Nonexpansion States, Excluding Montana and Louisiana (n = 1184 Person-Years) eReference. [file jamahealthforum-e245025-s001.pdf]

Supplementary Online Content

Tipirneni R, Roberts ET, Levy HG, et al. Changes in utilization and costs for older adults aging into Medicare after the Affordable Care Act. *JAMA Health Forum*. Published online January 17, 2025. doi:10.1001/jamahealthforum.2024.5025

eMethods. Methodologic Appendix

eTable 1. Interrupted Time Series: Overall Changes in Health, Utilization, and Costs for Adults Aged 65 to 68 Years With Income Up to 400% FPL Before and After the Affordable Care Act, Excluding Early Expansion States (n = 3745 Person-Years)

eTable 2. Difference-in-Difference Analyses Comparing Changes for Adults Aged 65 to 68 Years With Income Up to 138% FPL in Medicaid Expansion and Nonexpansion States, Excluding Early Expansion States (n = 1074 Person-Years)

eTable 3. Interrupted Time Series: Overall Changes in Health, Utilization, and Costs for Adults Aged 65 to 68 Years With Income Up to 400% FPL Before and After the Affordable Care Act, Excluding Montana and Louisiana (n = 4359 Person-Years)

eTable 4. Difference-in-Difference Analyses Comparing Changes for Adults Aged 65 to 68 Years With Income Up to 138% FPL in Medicaid Expansion and Nonexpansion States, Excluding Montana and Louisiana (n = 1184 Person-Years)

eReference.

This supplementary material has been provided by the authors to give readers additional information about their work.

© 2025 Tipirneni R et al. *JAMA Health Forum*.

eMethods. Methodologic Appendix

Overall Study Sample Selection with Inclusion/Exclusion Criteria

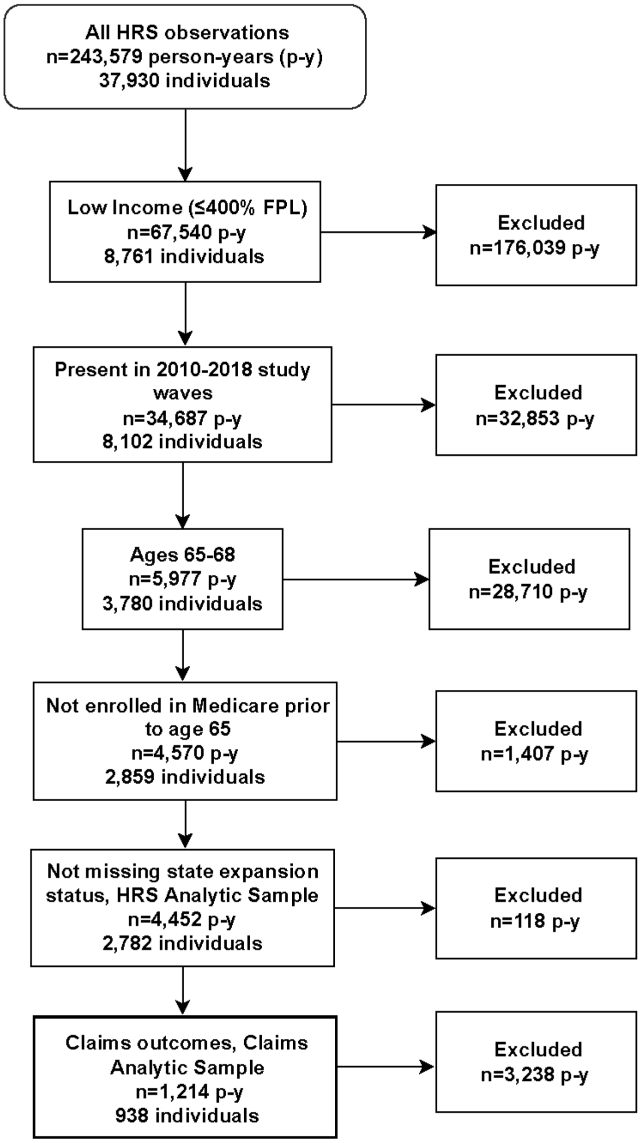

Study population: Treatment and Comparison Cohorts from Health and Retirement Study (HRS)

HRS participants were selected for inclusion based on potential exposure to the Affordable Care Act (ACA) coverage expansions (see table on next page). Individuals were included in study cohorts if their pre-age 65 income met eligibility thresholds for ACA coverage (≤400% FPL for any ACA coverage, ≤138% FPL for Medicaid expansion coverage, during ages 60-64). Treatment cohorts based on birth year and HRS study wave are shaded in blue while comparison cohorts are shaded in yellow. Treatment cohorts of 65-to-68-year-olds in 2016 and 2018 included individuals who were younger than 65 and thus eligible for ACA coverage expansions when they went into effect in 2014. Comparison cohorts of 65-to-68-year-olds in 2010 and 2012 included individuals who were all 65 or older by the time of ACA implementation in 2014. These comparison individuals did not have exposure to ACA coverage as they were already

© 2025 Tipirneni R et al. *JAMA Health Forum*.

eligible for Medicare prior to the 2014 ACA coverage expansions. Gray shading indicates a washout year for policy implementation.

Birth cohort	Birth year	HRS study wave (biennial survey)				
		2010	2012	2014	2016	2018
War Babies	1940	70	72	74	76	78
	1941	69	71	73	75	77
	1942	68	70	72	74	76
	1943	67	69	71	73	75
	1944	66	68	*70*	72	74
	1945	65	67	*69*	71	73
	1946	64	66	*68*	70	72
	1947	63	65	67	69	71
Early Baby Boomers	1948	62	64	66	68	70
	1949	61	63	65	67	69
	1950	60	62	64	66	68
	1951	59	61	63	65	67
	1952	58	60	62	64	66
	1953	57	59	61	63	65

Timing of observed survey and claims outcomes

For all cohorts, we used the first available HRS survey wave when participants were ages 65-68 to assess self-reported coverage, health, medication use, and out-of-pocket costs. For claims-based utilization outcomes, we examined linked Medicare data using a look-back period of 1 year using the first day of the month and year of their core survey interview as the index date.

Exposure classification: Medicaid expansion

We assessed exposure to ACA Medicaid expansion by comparing residents of states that did not expand Medicaid to states that expanded Medicaid as of Jan 1, 2018. States that had partial Medicaid eligibility expansions under non-ACA state authority before 2014 but not under ACA authority until 2014 (Arizona, Delaware, Hawaii, Massachusetts, New York, and Vermont) were classified as expansion states in our analysis. Six states that expanded Medicaid early under the ACA (California, Connecticut, District of Columbia, Minnesota, New Jersey, Washington) still experienced further enrollment increases after 2014 and were thus classified as expansion states. States classified as non-expansion states included Alabama, Florida, Georgia, Idaho, Kansas, Maine, Mississippi, Missouri, Nebraska, North Carolina, Oklahoma, South Carolina, South Dakota, Tennessee, Texas, Utah, Virginia, Wyoming. ACA Medicaid expansion states included Alaska, Arizona, Arkansas, California, Colorado, Connecticut, Delaware, D.C., Hawaii, Illinois, Indiana, Iowa, Kentucky, Louisiana, Maryland, Massachusetts, Michigan, Minnesota, Montana, Nevada, New Hampshire, New Jersey, New Mexico, New York, North Dakota, Ohio, Oregon, Pennsylvania, Rhode Island, Vermont, Washington, West Virginia. A timeline of state Medicaid expansion is included below:

© 2025 Tipirneni R et al. *JAMA Health Forum*.

Timeline of state Medicaid expansion implementation

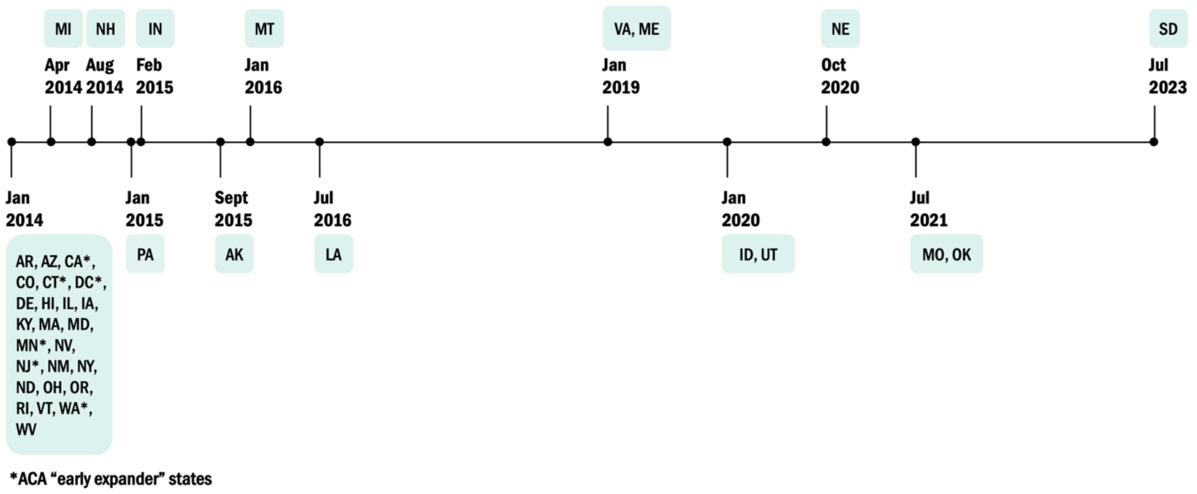

Identification of health care utilization outcomes from Medicare claims

Outpatient visits were extracted from the Outpatient and Carrier files and included claim lines with CPT codes for evaluation and management visits (99201, 99202, 99203, 99204, 99205, 99211, 99212, 99213, 99214, 99215, 99241, 99242, 99243, 99244, 99245) and for preventive health/health maintenance (99381, 99382, 99383, 99384, 99385, 99386, 99387, 99391, 99392, 99393, 99394, 99395, 99396, 99397). We excluded all emergency department visits with revenue center codes (0450, 0451, 0452, 0453, 0454, 0455, 0456, 0457, 0458, 0459, 0981) from the count of outpatient visits. Claim lines were converted into an event level file using the dates on the claims file to identify distinct non-emergency department outpatient visits.

Emergency department (ED) visits were extracted from the Outpatient and Inpatient files using revenue codes (0450-0459, 0981, as listed above) per guidance from ResDAC.[1] ED visits identified using Inpatient files were linked with those identified using Outpatient files to identify overlapping or adjacent ED visits and prevent double-counting in the case of ED visits that resulted in an admission.

Unique inpatient hospital admissions were determined using the Medicare Provider Analysis and Review (MedPAR) files.

Testing of parallel trends assumption in difference-in-differences analyses (DID)

Graphs below show pre-period (2010, 2012, 2014) trends in expansion vs. non-expansion states. To assess for parallel trends in the pre-period we also conducted analyses for all outcomes, interacting time with a dummy variable representing expansion state status, followed by a joint Wald test to assess the assumption that the interaction term was equal to zero. All Wald tests for the core survey outcomes were non-significant. For the claims outcomes, Wald tests for the outpatient visit and Medicare payment variables were significant, so the DID results for those analyses should be considered with some caution.

Pre-Period Trends in Medicaid Expansion and Non-expansion States across All Outcomes

© 2025 Tipirneni R et al. *JAMA Health Forum*.

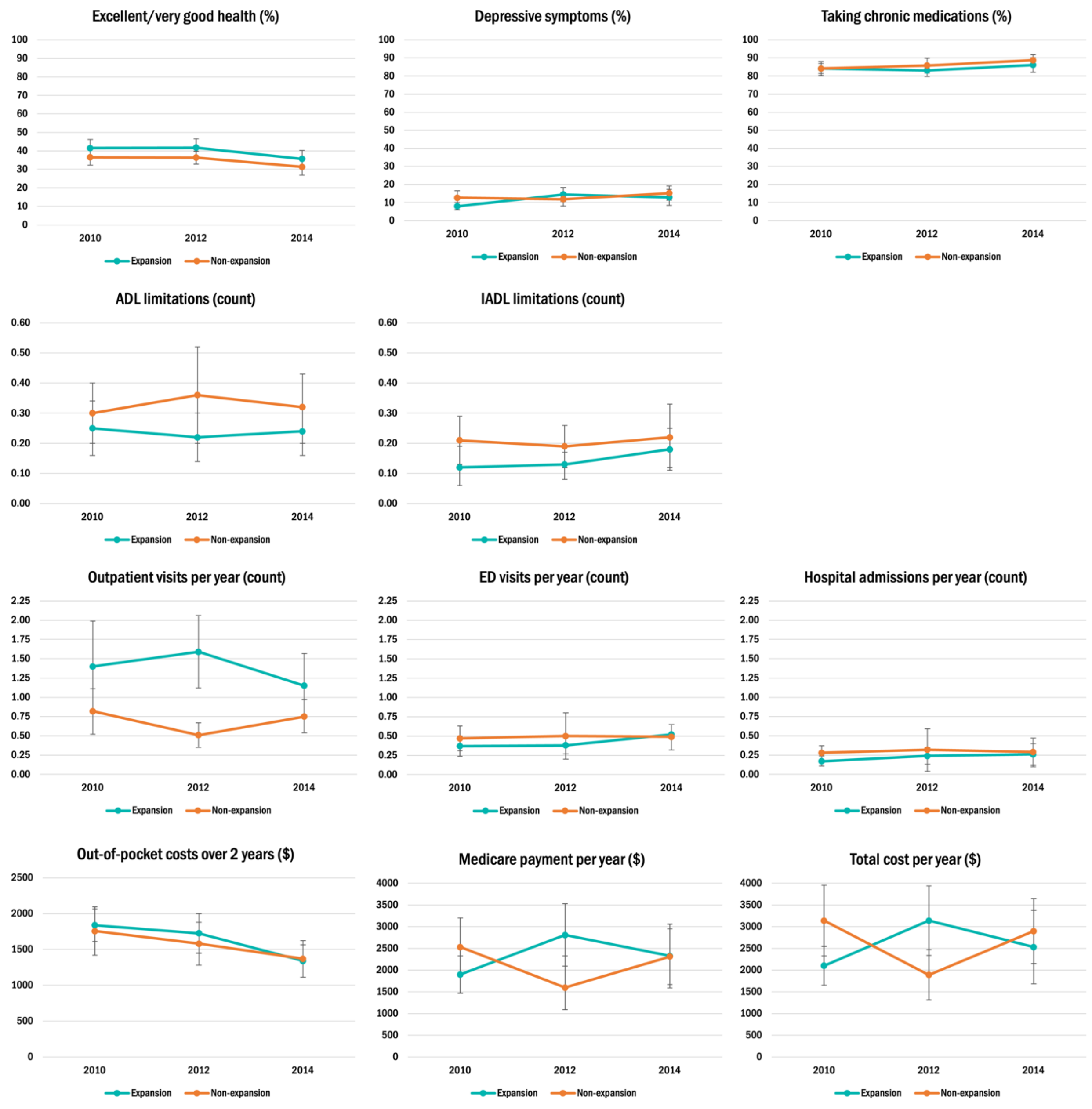

Contextual Analyses: Changes in Health Insurance Coverage for Middle-Aged Adults

To interpret the magnitude of health and utilization changes observed among new Medicare entrants (older adults ages 65-68) in the HRS in the context of preceding coverage changes for middle-aged adults, we analyzed American Community Survey (ACS) data for people ages 50-64 between 2013 (prior to the ACA's coverage expansions) and 2019 (following the ACA expansions and approximating the final year of the HRS analysis). We examined outcomes of any health insurance coverage (overall before and after the ACA, as well as comparing Medicaid expansion and non-expansion states) and Medicaid coverage (comparing Medicaid expansion

© 2025 Tipirneni R et al. *JAMA Health Forum*.

and non-expansion states). The ACS analyses demonstrated overall large coverage gains for middle-aged adults following the ACA, with somewhat larger gains in any health insurance coverage in non-expansion states and Medicaid coverage in expansion states:

Overall Changes in Any Health Insurance for Adults Aged 50-64 with Income Up to 400% FPL Before and After the Affordable Care Act

Outcome	Pre-ACA (2013)	Post-ACA (2019)	Delta (%)
Any health insurance	76.2%	86.1%	9.9%***

* significant at α = 0.1. ** significant at α = 0.05. *** significant at α = 0.01.

Difference-in-Difference Analysis Comparing Changes in Any Health Insurance for Adults Aged 50-64 with Income Up to 138% FPL in Medicaid Expansion and Non-Expansion States

	Non-expansion states			Expansion states			
	Pre-ACA (2013)	Post- ACA (2019)	Delta (%)	Pre- ACA (2013)	Post- ACA (2019)	Delta (%)	DID (%)
Any health insurance	57.1%	76.3%	19.2%***	72.2%	86.3%	14.1%***	-5.1%***

* significant at α = 0.1. ** significant at α = 0.05. *** significant at α = 0.01.

Difference-in-Difference Analysis Comparing Changes in Medicaid Coverage for Adults Aged 50-64 with Income Up to 138% FPL in Medicaid Expansion and Non-Expansion States

	Non-expansion states			Expansion states			
	Pre- ACA (2013)	Post- ACA (2019)	Delta (%)	Pre- ACA (2013)	Post- ACA (2019)	Delta (%)	DID (%)
Medicaid	24.6%	38.7%	14.2%***	42.0%	57.5%	15.6%***	1.4%***

* significant at α = 0.1. ** significant at α = 0.05. *** significant at α = 0.01.

© 2025 Tipirneni R et al. *JAMA Health Forum*.

SENSITIVITY ANALYSIS: DROPPING 6 ACA EARLY EXPANSION STATES

eTable 1. Interrupted Time Series: Overall Changes in Health, Utilization, and Costs for Adults Aged 65 to 68 Years With Income Up to 400% FPL Before and After the Affordable Care Act, Excluding Early Expansion States (n = 3745 Person-Years)

**Outcome**	**Pre ACA (2012) (95% CI)**	**Post ACA (2018) (95% CI)**	**Pre/Post change (95% CI)[c]**	***p* value[c]**
**Health status[a]**				
Excellent/very good health (%)	40.8 (37.2, 44.4)	41.6 (37.7, 45.6)	0.8 (-4.4, 6.0)	0.75
Depressive symptoms (%)	12.9 (9.5, 16.3)	10.5 (7.1, 13.8)	-2.4 (-6.9, 2.1)	0.29
Activities of daily living (ADL) limitations (count)	0.3 (0.2, 0.3)	0.3 (0.2, 0.4)	0.0 (-0.1, 0.4)	0.72
Instrumental activities of daily living (IADL) limitations (count)	0.2 (0.1, 0.2)	0.2 (0.1, 0.2)	0.0 (-0.1, 0.1)	0.66
**Utilization[b]**				
Taking chronic medications (%)	85.7 (82.1, 89.2)	79.8 (76.0, 83.5)	**-5.9 (-10.9, -0.9)**	**0.02**
Total number of outpatient visits per year (count)	1.2 (0.7, 1.7)	0.9 (0.6, 1.3)	-0.3 (-0.9, 0.4)	0.40
Total number of emergency department visits per year (count)	0.5 (0.3, 0.8)	0.5 (0.3, 0.7)	-0.1 (-0.3, 0.2)	0.67
Total number of hospital admissions per year (count)	0.4 (0.2, 0.5)	0.1 (0.1, 0.2)	**-0.2 (-0.4, -0.01)**	**0.04**
**Out-of-pocket costs over 2 years ($)[a]**	1,706 (1,474, 1,939)	1,221 (1,056, 1,387)	**-485 (-784, -186)**	**0.002**
**Medicare Costs[b]**				
Medicare payment per year ($)	2,441 (1,740, 3,142)	1,791 (1,109, 2,474)	-650 (-1,737, 437)	0.24
Total cost including beneficiary and Medicare payments per year ($)	2,762 (1,978, 3,546)	2,101 (1,379, 2,824)	-661 (-1,786, 464)	0.24

Source: Authors' analysis of Health and Retirement Study core survey data and linked fee-for-service Medicare claims data.

Notes: Interrupted time series analysis was used to assess overall changes before compared with after the Affordable Care Act's insurance coverage expansions in 2014. Changes represent the difference between the baseline year (2012) and the final year available for analysis (2018). All analyses were survey-weighted and adjusted for gender, race/ethnicity, education level, marital status, and assets, as well as year fixed effects.

[a]Health and Retirement Study core survey data

[b]Linked fee-for-service Medicare claims data

[c]Bolded numbers indicate significant change at the p<.05 level.

© 2025 Tipirneni R et al. *JAMA Health Forum*.

eTable 2. Difference-in-Difference Analyses Comparing Changes for Adults Aged 65 to 68 Years With Income Up to 138% FPL in Medicaid Expansion and Nonexpansion States, Excluding Early Expansion States (n = 1074 Person-Years)

	**Expansion states[c]**			**Non-expansion states[c]**				
**Outcome**	**Pre ACA (2012)**	**Post ACA (2018)**	**Pre/Post change**	**Pre ACA (2012)**	**Post ACA (2018)**	**Pre/Post change**	**DID (95% CI)[c]**	***p* value[c]**
**Health status[a]**								
Excellent/very good health (%)	27.9 (19.4, 36.5)	41.0 (33.8, 48.2)	**13.1 (2.3, 23.9)**	34.0 (24.4, 43.6)	33.4 (26.3, 40.6)	-0.6 (-11.2, 10.1)	13.7 (-0.9, 28.2)	0.06
Depressive symptoms (%)	19.20(9.73 28.7)	15.0 (7.2, 22.8)	-4.2 (-16.7, 8.2)	17.2 (8.6, 25.8)	19.0 (8.9, 29.1)	1.8 (-8.8, 12.4)	-6.0 (-19.1, 7.0)	0.36
Activities of daily living (ADL) limitations (count)	04 (0.2, 0.6)	0.3 (0.2, 0.5)	0.0 (-0.3, 0.2)	0.4 (0.2, 0.6)	0.7 (0.4, 1.0)	0.3 (-0.1, 0.7)	-0.3 (-0.7, 0.1)	0.09
Instrumental activities of daily living (IADL) limitations (count)	0.2 (0.1, 0.3)	0.2 (0.1, 0.3)	0.0 (-0.2, 0.1)	0.3 (0.1, 0.4)	0.4 (0.3, 0.6)	0.2 (-0.1, 0.4)	-0.2 (-0.5, 0.1)	0.12
**Utilization[b]**								
Taking chronic medications (%)	80.8 (71.5, 90.1)	78.3 (70.2, 86.4)	-2.5 (-14.2, 9.2)	87.6 (81.4, 93.7)	86.1 (80.5, 91.6)	-1.5 (-10.4, 7.4)	-1.0 (-13.3, 11.3)	0.87
Total number of outpatient visits per year (count)	1.5 (0.9, 2.2)	1.7 (0.7, 2.7)	0.2 (-1.2, 1.5)	1.4 (0.5, 2.4)	1.1 (0.7, 1.5)	-0.3 (-1.2, 0.5)	0.5 (-1.0 1.9)	0.50
Total number of emergency department visits per year (count)	0.3 (0.1, 0.6)	0.9 (0.4, 1.3)	**0.5 (0.1, 1.0)**	0.6 (0.0 1.2)	0.4 (0.2, 0.7)	-0.2 (-0.9, 0.5)	0.7 (-0.1, 1.5)	0.08
Total number of hospital admissions per year (count)	0.2 (0.1, 0.3)	0.2 (0.1, 0.3)	0.0 (-0.2, 0.2)	0.6 (-0.3, 1.4)	0.2 (0.0, 0.3)	-0.4 (-1.2, 0.4)	0.4, (-0.4, 1.2)	0.30

© 2025 Tipirneni R et al. *JAMA Health Forum.*

**Out-of-pocket costs over 2 years ($)[a]**	1,373 (882, 1,864)	1,383 (1,025, 1,740)	9 (-542, 561)	2,183 (1,67, 2,708)	1,087 (787, 1,388)	**-1,095 (-1,740, -450)**	**1,105 (367, 1,843)**	**0.004**
**Medicare Costs[b]**								
Medicare payment per year ($)	2,720 (1,177, 4,263)	3,182 (1,367, 4,996)	462 (-1,842, 2,766)	3,261 (1,518, 5,003)	3,391 (1,441, 5,340)	130 (-2,381, 2,641)	332 (-2,765, 3,428)	0.83
Total cost including beneficiary and Medicare payments per year ($)	3,042 (1,335, 4,748)	3,350 (1,465, 5,236)	309 (-2,133, 2,750)	3,587 (1,640, 5,534)	3,418 (1,406, 5,429)	-169 (-2,801, 2,462)	478 (-2,783, 3,738)	0.77

Source: Authors' analysis of Health and Retirement Study core survey data and linked fee-for-service Medicare claims data.

Notes: Difference-in-difference analyses were used to compare changes in states that expanded Medicaid under the Affordable Care Act as of January 1, 2018, to states that did not expand Medicaid. All analyses were survey-weighted and adjusted for gender, race/ethnicity, marital status, education level, and assets, as well as year fixed effects.

[a]Health and Retirement Study core survey data

[b]Linked fee-for-service Medicare claims data

[c]Bolded numbers indicate significant change at the p<.05 level.

© 2025 Tipirneni R et al. *JAMA Health Forum*.

SENSITIVITY ANALYSIS: EXCLUDING 2016 EXPANSION STATES (MONTANA & LOUISIANA)

eTable 3. Interrupted Time Series: Overall Changes in Health, Utilization, and Costs for Adults Aged 65 to 68 Years With Income Up to 400% FPL Before and After the Affordable Care Act, Excluding Montana and Louisiana (n = 4359 Person-Years)

Outcome	Pre ACA (2012) (95% CI)	Post ACA (2018) (95% CI)	Pre/Post change (95% CI)[c]	*p* value[c]
**Health status[a]**				
Excellent/very good health (%)	41.6 (37.6, 45.5)	42.0 (38.2, 45.8)	0.5 (-4.7, 5.6)	0.86
Depressive symptoms (%)	12.4 (9.2, 15.7)	10.2 (7.1, 13.3)	-2.3 (-6.6, 2.1)	0.30
Activities of daily living (ADL) limitations (count)	0.3 (0.29, 0.4)	0.3 (0.2, 0.4)	0.0 (-0.1, 0.2)	0.63
Instrumental activities of daily living (IADL) limitations (count)	0.2 (0.1, 0.3)	0.2 (0.1, 0.2)	0.0 (-0.1, 0.03)	0.29
**Utilization[b]**				
Taking chronic medications (%)	84.3 (81.0, 87.7)	79.6 (76.0, 83.1)	**-4.7 (-9.5, -0.04)**	**0.05**
Total number of outpatient visits per year (count)	1.2 (0.8, 1.6)	0.8 (0.5, 1.2)	-0.4 (-0.9, 0.2)	0.17
Total number of emergency department visits per year (count)	0.5 (0.2, 0.7)	0.4 (0.2, 0.6)	-0.1 (-0.3, 0.2)	0.54
Total number of hospital admissions per year (count)	0.3 (0.2, 0.5)	0.1 (0.1, 0.2)	**-0.2 (-0.4, -0.03)**	**0.03**
**Out-of-pocket costs over 2 years ($)[a]**	1,654 (1,434, 1,875)	1,226 (1,059, 1,394)	**-428 (-706, -150)**	**0.003**
**Medicare Costs[b]**				
Medicare payment per year ($)	2,490 (1,712, 3,267)	1,741 (1,099, 2,329)	-776 (-1,829, 278)	0.15
Total cost including beneficiary and Medicare payments per year ($)	2,806 (1,956, 3,655)	2,042 (1,386, 2,698)	-764 (-1,860, 331)	0.19

Source: Authors’ analysis of Health and Retirement Study core survey data and linked fee-for-service Medicare claims data.

Notes: Interrupted time series analysis was used to assess overall changes before compared with after the Affordable Care Act’s insurance coverage expansions in 2014. Changes represent the difference between the baseline year (2012) and the final year available for analysis (2018). All analyses were survey-weighted and adjusted for gender, race/ethnicity, education level, marital status, and assets, as well as year fixed effects.

[a]Health and Retirement Study core survey data

[b]Linked fee-for-service Medicare claims data

[c]Bolded numbers indicate significant change at the p<.05 level.

© 2025 Tipirneni R et al. *JAMA Health Forum*.

eTable 4. Difference-in-Difference Analyses Comparing Changes for Adults Aged 65 to 68 Years With Income Up to 138% FPL in Medicaid Expansion and Nonexpansion States, Excluding Montana and Louisiana (n = 1184 Person-Years)

	**Expansion states**			**Non-expansion states[c]**				
**Outcome**	**Pre ACA (2012)**	**Post ACA (2018)**	**Pre/Post change**	**Pre ACA (2012)**	**Post ACA (2018)**	**Pre/Post change**	**DID (95% CI)[c]**	***p* value[c]**
**Health status[a]**								
Excellent/very good health (%)	31.6 (23.5, 39.7)	38.5 (32.2, 44.9)	6.9 (-2.1, 16.0)	31.7 (22.4, 41.1)	34.6 (27.3, 41.9)	2.8 (-7.6, 13.3)	4.1 (-8.4, 16.6)	0.51
Depressive symptoms (%)	19.8 (10.5, 29.2)	14.1 (7.8, 20.3)	-5.7 (-17.3, 5.9)	17.3 (8.6, 25.9)	17.6 (7.8, 27.4)	0.3 (-9.8, 10.5)	-6.1 (-18.7, 6.5)	0.34
Activities of daily living (ADL) limitations (count)	0.6 (0.2, 0.9)	0.4 (0.2, 0.6)	-0.2 (-0.5, 0.2)	0.4 (0.2, 0.6)	0.6 (0.3, 0.9)	0.3 (-0.1, 0.6)	**-0.4 (-0.8, -0.02)**	**0.04**
Instrumental activities of daily living (IADL) limitations (count)	0.3 (0.1, 0.5)	0.2 (0.1, 0.3)	-0.1 (-0.2, 0.1)	0.3 (0.1, 0.5)	0.4 (0.3, 0.6)	0.2 (-0.1, 0.4)	-0.2 (-0.5, 0.1)	0.12
**Utilization[b]**								
Taking chronic medications (%)	80.8 (72.6, 89.0)	76.4 (69.4, 83.4)	-4.3 (-14.5, 5.8)	87.0 (80.8, 93.2)	85.3 (79.3, 91.4)	-1.7 (-10.7, 7.4)	-2.7 (-13.2, 7.8)	0.61
Total number of outpatient visits per year (count)	1.2 (0.6, 1.7)	1.3 (0.4, 2.1)	0.1 (-1.0, 1.2)	12.0 (0.6, 3.3)	1.2 (0.7, 1.7)	-0.8 (-2.1, 0.5)	0.9 (-0.4, 2.2)	0.19
Total number of emergency department visits per year (count)	0.4 (0.1, 0.6)	0.7 (0.4, 1.1)	0.4 (-0.1, 0.8)	0.6 (0.0 1.1)	0.3 (0.1, 0.6)	-0.2 (-0.8, 0.4)	0.6 (-0.1, 1.3)	0.07
Total number of hospital admissions per year (count)	0.2 (0.1, 0.3)	0.2 (0.1, 0.3)	0.0 (-0.2, 0.1)	0.6 (-0.8, 2.0)	0.2 (-0.1, 0.4)	-0.5 (-1.7, 0.8)	0.4, (-0.8, 1.7)	0.49

**Out-of-pocket costs over 2 years ($)[a]**	1,439 (1,000, 1,879)	1,277 (966, 1,588)	-162 (-643, 318)	2,158 (1,648, 2,667)	1,092 (794, 1,390)	**-1,065 (-1,703, -428)**	**903 (270, 1,536)**	**0.006**
**Medicare Costs[b]**								
Medicare payment per year ($)	2,635 (1,069, 4,201)	3,065 (1,426, 4,703)	430 (-1,816, 2,676)	2,786 (1,134, 4,438)	3,576 (1,564, 5,58)	790 (-1,780, 3,359)	-360 (-3,572, 2,852)	0.82
Total cost including beneficiary and Medicare payments per year ($)	2,907 (1,190, 4,624)	3,231 (1,501, 4,960)	324 (-2,076, 2,724)	3,061 (1,222, 4,899)	3,632 (1,554, 5,710)	571 (-2,117, 3,260)	-248 (-3,622, 3,127)	0.88

Source: Authors' analysis of Health and Retirement Study core survey data and linked fee-for-service Medicare claims data.

Notes: Difference-in-difference analyses were used to compare changes in states that expanded Medicaid under the Affordable Care Act as of January 1, 2018, to states that did not expand Medicaid. All analyses were survey-weighted and adjusted for gender, race/ethnicity, marital status, education level, and assets, as well as year fixed effects.

[a]Health and Retirement Study core survey data

[b]Linked fee-for-service Medicare claims data

[c]Bolded numbers indicate significant change at the p<.05 level.

© 2025 Tipirneni R et al. *JAMA Health Forum*.

eReference.

1. Barosso G. How to identify hospital claims for emergency room visits in the Medicare claims data [Internet]. Research Data Assistance Center (ResDAC). 2015. Available from: https://www.resdac.org/articles/how-identify-hospital-claims-emergency-room-visits-medicare-claims-data

© 2025 Tipirneni R et al. *JAMA Health Forum*.
