# Supplementary material for: Health Care Utilization and Costs for Older Adults Aging Into Medicare After the Affordable Care Act
Source: JAMA Health Forum. 2025 Jan 17;6(1):e245025. doi: 10.1001/jamahealthforum.2024.5025 (PMC11742520; doi:10.1001/jamahealthforum.2024.5025)
Supplement: Supplement 2. — Data Sharing Statement [file jamahealthforum-e245025-s002.pdf]

Data Sharing Statement

Tipirneni. Health Care Utilization and Costs for Older Adults Aging Into Medicare After the Affordable Care Act. *JAMA Health Forum*. Published January 17, 2025.
doi:10.1001/jamahealthforum.2024.5025

Data
Data available: No

Additional Information
Explanation for why data not available: The Health and Retirement Study (HRS) linked Medicare claims data are restricted data accessed by the study team under a Data Use Agreement. The authors would be happy to provide the analytic code used to construct the study cohorts and conduct the analyses upon request.
